# Checkpoint Inhibitors and the Changing Face of the Relapsed/Refractory Classical Hodgkin Lymphoma Pathway

**DOI:** 10.1007/s11912-022-01292-2

**Published:** 2022-06-13

**Authors:** Xiao-Yin Zhang, Graham P. Collins

**Affiliations:** 1 Department of Clinical Haematology, Oxford University Hospitals NHS Foundation Trust, Oxford, UK; 2 Department of Haematology, Cancer and Haematology Centre, Oxford University Hospitals NHS Foundation Trust, Oxford, OX3 7LE UK

**Keywords:** Hodgkin lymphoma, Relapsed/refractory, Checkpoint inhibitors

## Abstract

**Purpose of Review:**

Checkpoint inhibitors (CPIs) targeting PD1 are highly active in relapsed/refractory classical Hodgkin lymphoma. A plethora of recent studies, often small and non-randomised, have raised many questions about how to optimally integrate these into clinical practice. We aim to discuss the use of CPIs in different relapsed/refractory settings in an effort to better define their role and highlight areas of research.

**Recent Findings:**

CPIs have shown efficacy at first relapse, as salvage pre- and post-autologous (ASCT) and allogeneic stem cell transplant (alloSCT) and as maintenance post-ASCT. Immune-related adverse events require careful attention, especially when used peri-alloSCT, where it is associated with hyperacute graft-versus-host disease. Newer PD1 inhibitors, as well as strategies to overcome CPI resistance, are being tested.

**Summary:**

CPIs are increasingly deployed at earlier points in the classical Hodgkin lymphoma pathway. Whilst progress is clearly being made, randomised studies are required to more clearly define the optimal positioning of these agents.

## Introduction

Classical Hodgkin lymphoma (cHL) is a highly curable disease. Nevertheless, around a quarter of patients do not respond to, or later relapse after, conventional front-line therapy [1–4]. In recent years, novel agents such as brentuximab vedotin (BV) and checkpoint inhibitors (CPIs) have been added to the armamentarium of therapeutic agents for relapse/refractory (R/R) disease. CPIs are particularly active in cHL, resulting in high rates of durable remissions, and have been studied at various points throughout the disease pathway, from initial therapy to post-transplant consolidation. However, the optimal setting in which to use these agents remains unclear. This review seeks to discuss the use of CPIs in different relapsed/refractory settings in an effort to better define their role and highlight areas of research.

## Relapse Following ASCT — the Initial Indication

ASCT remains the standard of care for adult patients with R/R cHL based on two small and potentially outdated studies, demonstrating improved progression-free survival (PFS), but not overall survival (OS) over combination chemotherapy alone [5, 6]. Durable remissions are achieved in approximately half of patients [7], although the quality of remission prior to ASCT is a key determining factor of outcome [8]. Historically, the outcome for patients relapsing after an ASCT was poor with reported survival at 5 years of 30–35% [9–12], prompting the development of new therapeutics in the post-ASCT setting. In a pivotal phase 2 study, BV produced an overall response rate (ORR) of 75%, complete response (CR) rate of 34% and a median PFS of 5.6 months in this setting [13]. Whilst durable responses were seen in those who achieved CR (5-year OS and PFS of 64% and 52%, respectively), most patients require additional treatment within a year [14].

Nivolumab and pembrolizumab have demonstrated impressive activity in the post-ASCT setting, with an ORR of ~70% (mostly partial responses [PR]) and favourable toxicity profile in phase I and II trials [15, 16, 17•, 18•, 19, 20, 21•, 22]. In CheckMate-205, 243 patients divided in 3 cohorts — BV naïve (cohort A, 63 patients), BV after ASCT (cohort B, 80 patients) and BV before and/or after ASCT (cohort C, 100 patients) — were treated with nivolumab until disease progression or unacceptable toxicity [22–24]. Nivolumab could be discontinued after 1 year of CR in cohort C, and restarted if relapse occurs within 2 years. With extended follow-up (median 18 months), durable responses were seen in all three cohorts, with the overall ORR, CR rate and median duration of response (DOR) being 69%, 16% and 16.6 months, respectively. The median PFS was 14.7 months, which compared favourably to BV (5.6 months). Response was rapid, with a median time to initial response of 2.1 months.

Similar efficacy was seen with pembrolizumab in KEYNOTE-087 [19, 20]. Cohort 1 (*n* = 69) and cohort 3 (*n* = 60) received pembrolizumab for up to 2 years after relapse following ASCT (with or without BV post-ASCT). After 5-year extended follow-up, the ORR was 84.1% (cohort 1) and 68.3% (cohort 3), with median PFS of 16.4 months (cohort 1) and 19.7 months (cohort 3). In the total study population, the CR rate was 27.6% and median DOR 16.6 months. Of those who achieved CR on the trial (58 patients, 10 who underwent allogeneic stem cell transplant [alloSCT]), achieved very durable responses with median PFS of 56.5 months and 5-year PFS of 44.3%.

CPIs have an acceptable safety profile, with the majority of adverse events (AEs) being low grade. The most common grade 3–4 treatment-related AEs (TRAEs) were lipase increase (5%), neutropenia (3%) and raised alanine transaminase (3%) for nivolumab, neutropenia (2.4%) and diarrhoea (1.4%) for pembrolizumab. The majority of immune-related AEs (irAEs) were low grade, with the thyroid being the most commonly affected, plus also pneumonitis, hepatitis and rash. Only 7% TRAEs led to treatment discontinuation.

Based on these results, nivolumab and pembrolizumab were approved by the US Food and Drug Administration (FDA) and the European Medicines Agency (EMA) for use in R/R cHL in the post-ASCT setting.

## Consolidation Following ASCT

Only about half of patients will attain durable disease control after ASCT. The AETHERA study demonstrated that early post-ASCT consolidation with BV reduces relapse in high-risk R/R patients (HR = 0.57) compared to placebo (median PFS 42.9 vs. 24.1 months) [25], albeit with a risk of peripheral neuropathy (56% vs. 16%). Following on the success of this approach, the safety and efficacy of post-ASCT consolidation with CPI alone or in combination with BV were studied in two small phase 2 trials. Thirty patients with high-risk R/R cHL (90% ≥1 and 40% ≥2 modified AETHERA risk factors: primary refractory disease, relapsed within 12 months of frontline therapy, residual fluorodeoxyglucose [FDG]-avid disease after salvage, >1 salvage regimen needed to achieve remission, extranodal disease/B symptoms at relapse) received up to 8 cycles of pembrolizumab within 21 days of discharge (<60 days post-stem cell infusion) post-ASCT (median 34 days) [21•]. Ninety-three percent were in CR prior to ASCT and those that required >2 lines of relapse treatment were excluded. Prior CPI (20%) or BV (20%) were permitted if entered remission without intervening relapse. The 18-month PFS was 81% overall and similar in those considered high risk (≥2 risk factors PFS = 83%, eligible for the AETHERA trial PFS = 85%). Five patients relapsed by 18 months, at a median of 6 months. This compared favourably to those who received BV consolidation in the AETHERA trial (estimated 2-year PFS 63%). AEs were frequent but judged acceptable. Seventy-seven percent completed all 8 cycles of pembrolizumab. Of the 7 patients who did not, 4 were due to toxicity. Grade ≥3 TRAEs were reported in 27%. Grade ≥2 irAEs were reported in 40%, the most common of which was pneumonitis.

Following on from this, 59 patients were consolidated with 8 cycles of nivolumab and BV, at a median time of 54 days post-ASCT [26]. This was a similarly high-risk population (100% ≥1 and 64% ≥2 modified AETHERA risk factors, 51% prior BV, 42% prior CPI). The estimated 18-month PFS was impressive, at 95% overall, 92% and 89% in those with >2 and >3 risk factors, respectively. Only 49% patients completed all 8 cycles of both drugs (76% completed 8 cycles of one drug). Of those who discontinued both, 6/14 were due to AEs. Similar numbers discontinued BV (14%) and nivolumab (12%). The most common AEs were peripheral neuropathy (51%), neutropenia (42%), fatigue (37%) and diarrhoea (29%). irAEs were reported in 31%.

Current data suggest CPI consolidation following ASCT is effective with a significant toxicity signal that could limit its widespread adoption. Given the caveats of cross-trial comparisons, direct randomised comparisons are needed to determine the relative efficacy and toxicity of BV, CPI and combination consolidation post-ASCT.

## Moving CPI Therapy into the Pre-ASCT Setting

With the success of CPIs in the post-ASCT setting, earlier use in the disease course may enable more patients to be eligible for ASCT consolidation with subsequent improved outcomes. Pembrolizumab is the only CPI approved for use pre-ASCT, based on results from cohort 2 of the KEYNOTE-087 trial, a group of patients who relapsed post-BV but were ineligible for ASCT due to chemoresistance. All 81 patients in this cohort had R/R disease after ≥ 3 lines of prior therapy including BV. After extended 3-year follow-up, the ORR, CR and median DOR of this cohort were 66.7%, 25.9% and 11.1 months, respectively [27]. For those who achieved CR, response was durable with median DOR of 19.2 months. Median PFS was 11.1 months. Two patients went on to receive a stem cell transplant. Pembrolizumab thus represents a valuable treatment option in this difficult-to-treat patient group.

**Fig. 1:**
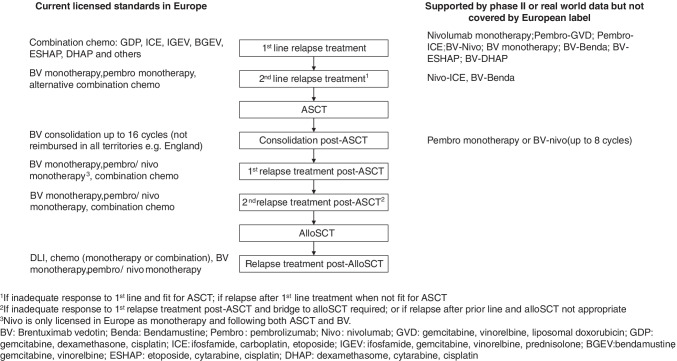
Various timepoints CPI can be used in the relapsed/refractory setting

The randomised phase III KEYNOTE-204 trial recently provided data on the relative efficacy and safety of pembrolizumab versus BV salvage pre-ASCT [28•]. Even though patients who had prior ASCT were included in the study, 63% of the 300 patients were ineligible for ASCT due to chemoresistance (44%), age (9%) and comorbidities (1%). Only ~5% patients had prior BV exposure (but not resistance). Interim analysis reported a clinically meaningful superior PFS with pembrolizumab treatment compared to BV in those ineligible for ASCT (median PFS 12.5 vs. 5.7 months, HR 0.61). In the whole study population, the ORRs were 65.6% for pembrolizumab and 54.2% for BV (*p* = 0.023, non-significant) and the CR rates were similar (24.5% versus 24.2%). A longer median DOR was observed for pembrolizumab (20.7 months) than BV (13.8 months). Overall, 64 patients subsequently underwent ASCT and seven underwent alloSCT without previous ASCT. Pembrolizumab and BV were associated with a similar incidence of grade 3–4 TRAEs (19.6% vs 25%). Higher incidence of irAEs (hypothyroidism [15.5% vs 1.3%] and pneumonitis [10.8% vs 2.6%]) was observed with pembrolizumab, whereas BV was associated with more nausea (13.2% vs 4.1%) and peripheral neuropathy (18.4% vs 2%). On the basis of this trial, pembrolizumab was granted FDA approval for R/R cHL in general and is now licensed for use in Europe for patients whose disease has progressed after receiving at least 2 lines of therapy when ASCT is not a treatment option (in addition to its indication in those relapsed following ASCT) (Figure 1).

The question remains whether to use a CPI or BV as third-line therapy. Data on the optimal timing and sequencing of CPI and BV remains limited and should be the focus of future research. In those where ASCT is never a treatment option due to age and/or comorbidities, it makes sense to use CPI for its longer PFS, and reserving BV for those whose disease progressed after CPI therapy or for those with contra-indications to CPI use. For those who may be bridged to ASCT however, there is still doubt as to which agent (if any) is associated with better outcomes following ASCT. It would seem reasonable to use either agent initially, reserving the other for use in those who require further treatment pre-transplant

Limited data exists for re-treating patients who have discontinued CPI therapy in CR. In the Keynote 087 study, high rates of durable response were seen in the 20 patients who received a second course of pembrolizumab when relapsed after discontinuation (ORR 73.7%, median DOR 15.2 months) [20]. A French study reported 7 patients re-treated with nivolumab after it was initially discontinued due to either prolonged remission or toxicity. All 7 patients responded with 4 achieving a CR and 3 a PR [29]. In a small single institution study of 23 patients who discontinued nivolumab in CR, 11 relapsed and 9 were re-treated with single agent nivolumab. Three achieved a CR, 3 a PR and 3 an indeterminate response [30]. There is clear efficacy therefore in re-treating patients with PD1 inhibitor if stopped due to a CR. There is currently no data on whether using a different PD1 inhibitor or indeed a PDL1 inhibitor in this situation is beneficial. Furthermore, for patients not responding to one CPI, it is unclear whether the use of a different CPI (for example a CTLA4 inhibitor) will lead to responses.

## CPI and Chemosensitivity

ASCT has traditionally been reserved for chemosensitive patients due to more favourable outcomes, particularly in those who achieve a complete metabolic response (CMR) on PET-CT. However, in the era of novel agents, this dogma is increasingly challenged. Studies in non-small cell lung cancer [31, 32] and in non-Hodgkin lymphoma (NHL) [33] suggest that CPI may have a chemosensitising effect that may be prolonged, and that is not solely determined by the depth of response to CPI itself. Emerging evidence in cHL also supports this, with improved chemo-responsiveness seen after CPI exposure in previously chemorefractory patients in small, retrospective studies. For example, Rossi et al reported an ORR of 66% and median PFS of 11 months in a group of 30 chemorefractory patients with unsatisfactory prior response (PR or worse) to CPI, who were then re-treated with chemotherapy (with or without CPI). Fifteen of the 30 patients were re-exposed to the same chemotherapy regimen which they have had previously, 6 of which were refractory. A cohort of 81 multiply R/R cHL patients (median 4 prior lines of therapy before CPI, 49% had prior stem cell transplant), who received subsequent therapy (44% single agent or combination chemotherapy) after discontinuing CPI due to disease progression (65%) or toxicity, achieved ORR of 62% and median DOR of 5.6 months, compared to 2.6 months to the line of therapy given prior to CPI (*p* = 0.0003). Higher ORR was seen in CPI responders (76%) compared to non-responders (43%) [34]. The mechanism underlying a chemosensitising effect is unclear. One hypothesis is that alteration of the tumour microenvironment (TME) may enable more effective chemotherapy activity potentially by removing pro-survival signals emanating from the TME.

Good outcome has been reported with ASCT following CPI therapy in chemorefractory patients, suggesting that demonstrating “chemosensitivity” prior to ASCT may not be necessary. Merryman et al retrospectively reviewed the outcome of 78 high-risk multiply R/R patients, who underwent ASCT after CPI therapy [35]. Chemorefractory disease was common in this cohort, with many considered poor ASCT candidates (41% had positive pre-ASCT PET-CT). The outcome was very favourable, with 18-month PFS of 75% in patients who had a positive pre-ASCT PET-CT. Despite a high rate of BV refractoriness in this cohort, the outcome is better than that observed for similarly high-risk patients in the AETHERA trial that did not receive BV maintenance (18-month PFS ~50% for entire cohort, 30–40% for patients with 2+ or 3+ risk factors) [36]. Multivariate analysis showed that lack of response to CPI therapy (HR, 10.2; *p* <0.001), but not pre-ASCT PET status (HR, 2.4; *p* = 0.13) or an interval of >20 weeks from CPI to ASCT (HR, 3.4*; p* = 0.063), was significant predictors of inferior PFS.

## CPI in First Relapse

At first relapse, a variety of regimens have conventionally been used, with no randomised comparison to guide practice. The efficacy of platinum- or gemcitabine-based chemotherapy appear similar, with ORR and CR rates ranging between 70 and 90%, and 50 and 75%, respectively [37–42]. Attaining a complete metabolic response (CMR) pre-ASCT is of critical prognostic significance [43, 44], and recent studies incorporating CPI into initial relapse therapy have aimed to improve CMR rate before ASCT. Removing traditional cytotoxic agents from regimens may also reduce long-term morbidities including secondary malignancies, cardiovascular disease and infertility, which are of particular significance given the relatively young age of R/R cHL patients.

### CPI, Chemotherapy and Radiotherapy

The addition of CPIs to conventional chemotherapy regimen resulted in promising efficacy in a small number of phase 2 studies to date. In a high-risk group of patients, pembrolizumab and GVD (gemcitabine, vinorelbine and liposomal doxorubicin) combination demonstrated high efficacy, good tolerability and high rate of transition to ASCT. Thirty-nine patients (41% primary refractory, 38% relapsed within a year of front-line therapy) treated with 2–4 cycles of pembrolizumab and GVD as first-line relapse therapy, followed by ASCT with or without BV maintenance at investigator’s discretion showed impressive ORR and CR rates, at 100% and 95%, respectively, with 92% patients in CR after 2 cycles and 95% proceeding directly to ASCT [45]. A third of the patients received post-ASCT BV maintenance. With a relatively short follow-up (median 13.5 months), all transplanted patients remain in remission. There were relatively few grade 3 AEs, and 13% patients received steroids for irAEs.

In a similar phase 2 study, 42 ASCT-eligible R/R patients (16 primary refractory) were treated with 3 cycles of pembrolizumab and ICE (ifosfamide, carboplatin, etoposide) chemotherapy plus an additional cycle of pembrolizumab monotherapy [46]. The ORR was 97.3%, with 86.5% in CMR post-2 cycles. Ninety-five percent patients proceeded to ASCT and the median PFS was 26.9 months with a median follow-up of 27 months. This combination appeared somewhat more toxic, with 52.3% patients developing grade 3/4 AEs, and there were two grade 5 AEs that were possibly related to pembrolizumab.

In a PET-adapted approach, 39 patients were given nivolumab, with those in CMR after 6 cycles proceeding to ASCT [47]. PET positive patients received 2 cycles of nivolumab and ICE. After 6 cycles of nivolumab, the ORR and CR rates were 90% and 77%, respectively. Seven patients received nivolumab and ICE, all of whom responded with 6 CRs. Tantalisingly, the majority of patients in this study underwent ASCT without prior cytotoxic chemotherapy at relapse, although longer follow-up is needed to demonstrate the durability of this strategy.

In the solid tumour setting, there is data that radiation could potentially sensitise to checkpoint inhibition via the induction of immunogenic cell death [48]. Whilst radiotherapy is a very effective modality for some patients with relapsed disease, the effect on CPI has not been extensively studied or reported.

Unsurprisingly, with CPIs’ high overall efficacy and potential chemosensitising effect, combining with chemotherapy resulted in potent efficacy in the first relapse setting. The optimal timing of CPI and chemotherapy (sequential versus combination), however, remains to be established.

### CPI and BV

With impressive single-agent activity, BV and CPI combination therapy is an obvious follow-on strategy that has been tested in an open-label phase I/II trial. Single agent BV, used in the first relapse setting, has a CR rate of 27 to 35%, with 27 to 48% able to proceed directly to ASCT [49–51]. In a phase 1/2 study, 61 R/R cHL patients whose disease progressed after first-line therapy were treated with 4 cycles of BV and nivolumab, followed by ASCT as per investigator’s discretion [52]. ORR and CR were 85% and 67%, respectively. Ninety-two percent proceeded to ASCT with 74% did so directly following completion of 4 cycles of BV and nivolumab, whilst the remainder did so following additional relapse therapy. Responses were durable, with estimated 3-year PFS of 77% overall, 91% in those who proceeded directly to ASCT with extended follow-up (median 34.3 months). Combination treatment was well tolerated, with 92% completing all 4 cycles (2 patients discontinued due to AEs). Most TRAEs were low grade, with nausea (52%) and infusion-related reactions (IRR) (43%) being most common. Grade 3/4 AEs included pneumonitis (3%), rash (1%), raised AST (1%), diarrhoea (1%) and Guillain-Barre syndrome (1%). Eighteen percent patients experienced irAEs requiring steroid therapy.

In this high-risk cohort enriched for primary refractory (44%) and early relapsed (30%) cHL, BV and nivolumab combination provided an effective bridge to ASCT in the majority of patients and was well tolerated, offering a “chemotherapy-free” alternative. Response rates appear to be superior in combination. A phase 2 trial (CheckMate-744) in 5 to 30 years old with R/R cHL is currently ongoing, evaluating a risk-stratified, response-adapted approach using nivolumab and BV and, for patients without CMR, BV and bendamustine. Preliminary results showed a promising CMR rate of 88% pre-ASCT, 59% after nivolumab and BV alone [53]. Randomised studies are needed to reliably determine comparative efficacy with established regimens and CPI-chemotherapy combinations.

## CPI Therapy and Allogeneic Stem Cell Transplant

### CPI Prior to alloSCT

Historically, patients who relapse after ASCT have poor prognosis, with median survival of 2 years or less [9–12]. Whilst CPIs have been a major breakthrough in this setting, it is clear from extended follow-up that the majority of patients will relapse within 1–2 years of starting CPI therapy [22, 23, 54]. AlloSCT remains the only treatment modality with curative potential, with 3-year relapse-free survival of 40% in those transplanted post-2000 in a meta-analysis of 38 studies (*n* = 1850) [55].

However, soon after the introduction of CPI therapy as a bridge to alloSCT, reports emerged relating to increased risk of fatal hyper-acute graft-versus-host-disease (GVHD), non-infectious febrile episodes requiring corticosteroids and other irAEs [56, 57]. This is perhaps unsurprising, as immune checkpoints are normal regulatory mechanisms that limit T-cell-mediated immune responses and maintains T-cell self-tolerance. With their long half-life, residual PD-1 inhibition in the peri-alloSCT period could potentially enhance donor T-cell responses, which could not only augment the graft-versus-lymphoma (GVL) effect, but also increase the incidence or severity of immune complications. Over the last 5 years, a number of small, retrospective studies reviewed the safety and efficacy of alloSCT in patients treated with CPI. A summary of these studies can be found in Table 1 [58–67].

**Table 1:** Summary of studies evaluating safety and efficacy of CPI therapy peri-alloSCT

Pre-/post-alloSCT	CPI	*n*	Median interval between CPI and alloSCT	GVHD prophylaxis	Stem cell source	aGVHD II–IV	aGVHD III–IV	cGVHD	irAEs	NRM	CIR	PFS/OS	Median follow-up	Reference
Pre-	Nivo (87%) Nivo+ipi (2%) Pembro (9%)	122	30, 42, 43, 44, 62 days or NR	22% PTCY	61% PBSC 16% BM 18% NR	NR	28%	26%	NR	15% (6 months)	NR	NR	NA	Dada et. al [58]
Pre-	Nivo	39	1.9 months (0.5–5.7)	51.3% PTCY	95% PBSC 5% BM	33%	5.1%	35%	35.9% SRNIFS	13.2% (1 year)	NR	69.4%/71.9% (2 years)	18.4 months (4.2–45.7)	Martinez et. al [65]
Pre-	Nivo (62%) Nivo+ipi (10%) Pembro (28%)	39	62 days (7–260)	36% PTCY	72% PBSC 28% BM	44% (1 year)	23% (1 year)	41% (1 year)	18% SRNIFS	11% (1 year)	14% (1 year)	76%/89% (1 year)	12 months (2–33)	Merryman et. al [59]
Pre-	NR	37	51 days (23–472)	100% PTCY	14% PBSC 84% BM	33% (1 year)	NR	3% (2 year)	NR	6% (3 years)	4% (3 years)	90%/94% (3 years)	15 months (1.4–45.9)	Paul et. al [62]
Pre-	Nivo	15	NR	NR	NR	3/15 (20%)	NR	3/15 (20%)	NR	1/15 (7%)	NR	78%/89% (2 years)	NR	Bekoz et. al [60]
Pre-	Nivo (57%) Pembro (14%) Ipi (21%)	14 (10 cHL)	42 days (18–212)	100% PTCY	14% PBSC 86% BM	6/14 (43%)	NR	0/14	NR	0%	NR	NR	10 months (6–12)	Schoch et. al [66]
Pre-	Nivo	6 (5 cHL, 1 NKTCL)	83.5 days (34–154)	50% PTCY	NR	50%	50%	0%	NR	50%	NR	NR	29 months (5–57)	Nieto et. al [67]
Pre-	Nivo (92%) Pembro (8%)	25	59 days (23–539)	32% PTCY	44% PBSC 48% BM	47.1% (100 day)	16.6% (100 day)	34.2% (1 year)	60% NIFS	8.4% (1 year)	27.9% (1 year)	63.7%/81.3% (1 year)	319 days (29–1346)	Ito et. al [63]
Post-	Nivo (95%) Pembro (5%)	20	589 days (49–3543)	10% PTCY	55% PBSC 30% BM	15.0% (100 day)	NR	30% (moderate/severe, 1 year)	30%	0%	47.1% (1 year)	52.6%/89.7% (1 year)	1717 days (681–4554)	Ito et. al [63]
Post-	Nivo	20	23 months (2––111)	NR	NR	6/20 (all grades)	NR	NR	NR	2/20	NR	58.2%/78.7% (1 year)	370 days (24–486)	Herbaux et. al [61]
Post-	Nivo (90%) Pembro (10%)	31 (29 cHL)	790 days (146–3289)	NR	94% PBSC	48%	19%	35%	NR	26%	NR	NR	428 days	Haverkos et. al (64)

Abbreviations: *alloSCT*, allogeneic stem cell transplant; *BM*, bone marrow; *cHL*, classical Hodgkin lymphoma; *CIR*, cumulative incidence of relapse; *CPI*, checkpoint inhibitor; *aGVHD*, acute graft versus host disease; *cGVHD*, chronic graft versus host disease; *ipi*, ipilimumab; *irAEs*, immune-related adverse events; *NIFS*, non-infectious febrile syndrome; *nivo*, nivolumab; *NKTCL*, NK-cell/T-cell lymphoma; *NR*, not reported; *NRM*, non-relapse mortality; *OS*, overall survival; *PBSC*, peripheral blood stem cell; *pembro*, pembrolizumab; *PFS*, progression-free survival; *PTCY*, post-transplant cyclophosphamide; *SRNIFS*, steroid-requiring non-infectious febrile syndrome

Merryman et al (2017) retrospectively reviewed the outcome of 39 patients (79% cHL, 21% NHL) previously treated with CPI at a median time of 62 days before alloSCT, using both related and unrelated donors and several different GVHD prophylactic regimens [18•]. Whilst the overall incidence of acute and chronic GVHD did not appear to differ significantly to expected, the incidence of grade 4 aGVHD appears higher than prior studies (13% vs 3–4%) [68–72]. Three out of 4 treatment-related deaths were from a particularly rapid form of aGVHD, which is rare after reduced-intensity conditioning (RIC) alloSCT. In addition, 7 patients (18%) developed a non-infectious febrile syndrome shortly after transplant requiring prolonged courses of steroids. Despite increased acute toxicity, the rate of non-relapse mortality (NRM) (11%) was not appreciably higher than previously published series in similar patient groups. CPI therapy may be associated with lower-than-expected relapse rate, as a lower 1-year cumulative incidence of relapse (CIR) (14%) was observed here compared to many historical series of cHL patients undergoing RIC alloSCT [68, 70]. However, such cross-study comparison is likely confounded by baseline differences in the study cohorts. The duration of time between the last dose of CPI and alloSCT (>60 days vs <60 days) did not significantly affect 1-year rates of OS, PFS, CIR, NRM or the incidence of GVHD or febrile syndrome. Patients who received bone marrow grafts (*n* = 11) appear to have had lower rates of grade 3–4 aGVHD compared to patients who received peripheral blood stem cells (*n* = 28) (0% vs 32%). Based on this limited evidence, the use of bone marrow grafts in the post-CPI setting may be preferable. There were no significant differences based on GVHD prophylaxis regimen in this study.

A larger pooled analysis of 6 studies comparing patients undergoing alloSCT after CPI (*n* = 122, 2015–2017) to those who were not exposed to CPI (*n* = 978, 2015–2018) [58] also reported a higher rate of grade 3–4 aGVHD in those with prior exposure to CPI (28% vs 8%, *p* = 0.02), but no significant difference in cGVHD or NRM at 6 months. Here, no association was found between the number of cycles of CPI prior to alloSCT or days from last administration of CPI to alloSCT and grade 3–4 aGVHD.

The effects of CPI appear to be long-lasting. In one study, nivolumab was detectable and its presence was associated with lower percentage of PD-1+ T-cells in the plasma of previously exposed patients post-alloSCT, despite a median time of 83.5 days since the last dose [67]. In another study, PD-1 expression was significantly decreased across all T-cell subsets up to 6 months post-alloSCT, despite a median time from last CPI therapy to transplant of 148 days [59]. The long-lasting effect of CPI therapy may explain the absence of any apparent association between the time interval from CPI therapy to transplantation and early toxicity. A period of delay of even several months may not mitigate the impact of CPI on alloSCT outcomes. However, despite the lack of supporting evidence, a working group recommended holding CPI therapy for 6 weeks before alloSCT and this remains common practice [73].

The prophylactic regimen used is well-known for influencing the frequency and severity of GVHD. Post-transplant cyclophosphamide (PTCY) has been a very effective prophylactic regimen for reducing GVHD in other transplant settings associated with an increased risk of GVHD [65]. Emerging data suggest that PTCY may reduce aGVHD in the post-CPI setting.

In a group of 25 patients who received CPI (median 9 cycles) at a median interval of 59 days prior to alloSCT, aGVHD was less frequent and less severe (100-day cumulative incidence of grade I–IV GVHD was 25% vs 88.2%, grade III–IV GVHD 0% vs 23.5%) in patients who received PTCY prophylaxis (*n* = 8) compared to those who did not (*n* = 17) [63]. A shorter interval (≤ 84 days) between the last dose of CPI and alloSCT (*p* = 0.11) and no PTCY use (*p* = 0.13) showed a non-significant association with higher risk of grade II–IV aGVHD. Nieto et al (2020) noted differential early T-cell response (more effector T-cell, higher proportion of IFN-gamma producing T-cells) after alloSCT in patients pre-exposed to nivolumab compared to those who did not. However, this was seen only when tacrolimus/sirolimus but not PTCY was given as GVHD prophylaxis, offering some mechanistic insight into the observation that PTCY prophylaxis is associated with lower incidence and severity of aGVHD.

Data to date point to an association between prior CPI exposure and increased incidence and severity of acute but not chronic GVHD post-alloSCT. The NRM appears unaffected and the CIR may be reduced. There is conflicting evidence whether the incidence and severity of aGVHD are affected by the time interval between CPI exposure and alloSCT, with some data suggesting that delaying alloSCT may reduce aGVHD, whilst others hint at a prolonged, long-term effect post-CPI exposure. There is limited evidence suggesting that PTCY prophylaxis may modify the early T-cell response post-transplant, and reduces the incidence and severity of aGVHD in those with prior CPI exposure. Larger studies are required to confirm this and to determine whether this strategy achieves the right balance between GVH and GVL effects.

### CPI Post-alloSCT

CPI treatment post-alloSCT, whilst effective (ORR ~70%), appears to be associated with the rapid development of aggressive treatment-emergent GVHD (teGVHD), even in those without a prior history of GVHD. In a group of 20 patients who received CPI (median 8 cycles,) at a median of 589 days post-alloSCT, teGVHD was observed in 50% patients, and irAE in 55%, which was considerably higher than that reported in non-alloSCT patients [63]. Fifty-five percent patients required treatment discontinuation due to teGVHD or irAEs. At the start of CPI therapy, 65% and 45% patients had a past history of aGVHD and cGVHD, respectively, but GVHD was active in only 5% and 20% patients, respectively.

When a group of 20 cHL patients who relapsed after alloSCT was given nivolumab (median 8 cycles), GVHD occurred in 6 patients (30%) a week after nivolumab initiation and 2 died as a result of GVHD [61]. All 6 patients had prior history of acute GVHD. Time between alloSCT and nivolumab treatment was significantly shorter in patients who presented with nivolumab-induced GVHD (median 8.5 months vs 28.5 months).

Finally, after a group of 31 lymphoma patients (30 cHL) were treated with CPI at a median of 2.2 years post-transplant, 17 patients (55%) developed GVHD (severe in 9/17) after a median of 1-2 cycles, including 5 with no prior history of GVHD. Only 2 of these 17 patients achieved CR to GVHD treatment, and 14 of 17 required ≥2 systemic therapies. There were 8 (26%) death related to new-onset GVHD after CPI therapy, including 5 associated with hepatic GVHD that is typically rare after alloSCT. Whilst effective, post-alloSCT CPI therapy should be used with caution, particularly in those with early relapse.

Given concern over the risk of severe aGVHD and other irAEs with alloSCT post-CPI therapy, patient selection is key in identifying those likely to benefit from alloSCT. Extended follow-up of the CheckMate-205 and KEYNOTE-087 trials have shown long-term remission in a sizeable minority of CPI-treated patients [23, 27]. The 3-year PFS was 18.8% in KEYNOTE-087, and the 5-year PFS was 18% in CheckMate-205. Twelve patients in cohort C of the CheckMate-205 trial stopped nivolumab after ≥ 1 year of CR. After a median of 48 months from last treatment, 6 patients remain in response: 3 were re-treated with nivolumab after disease progression, 2 achieving CR and 1 achieving PR [23]. In a cohort of 11 patients who discontinued nivolumab whilst in CR (including 7 patients who had prolonged remission [median treatment duration 13.8 months] and 4 patients who experienced toxicity [including 3 post-alloSCT)) [74], 80% remains in CR after a median follow-up of 21.2 months, with half having been off treatment for more than 21 months. The decision whether a patient responding to CPI should continue treatment, stop treatment or proceed to alloSCT consolidation remains a challenging one.

## Future Directions

### Restoration of Sensitivity CPI

With the current trend of incorporating CPIs at increasingly earlier point in the disease course, patients with R/R cHL will become increasingly resistant to CPIs. An interesting new area of development is the use of epigenetic modifying drugs — DNA hypomethylating agents (HMA) and histone deacetylase inhibitors (HDACi) to enhance the efficacy and restore sensitivity to CPIs. Several small cohort studies have shown that the addition of HMA or HDACi to CPI treatment increases CR rate. Combination therapy with camrelizumab (a new CPI) and decitabine led to a higher CR rate (71%) compared to camrelizumab monotherapy (23%) in a group of CPI naïve, Chinese patients with R/R cHL [75]. Falchi et al (2016) treated 10 multiply R/R cHL patients with CPI [76]. Five out of the 10 patients had prior exposure to azacitadine and romidepsin on a phase I clinical trial. All 5 azacitidine/romidepsin treated patients achieved CR, compared to 2 out of 4 patients without prior azacitidine/romidepsin exposure. Herrera et al (2019) treated 12 cHL patients with pembrolizumab and vorinostat. Eleven of the 12 patients had prior BV and 7 had prior CPI, including 3 who were refractory to CPI [77]. An impressive 100% ORR and 44% CR rate was seen in this cohort. These small studies suggest a synergistic effect between HMA/HDACi and CPI. Larger studies are required to explore their potential in the R/R cHL treatment algorithm.

### New CPIs

Multiple new PD-1 inhibitors are currently in development with preclinical studies showing structural differences to existing PD1 inhibitors. For example, tislelizumab binds to a unique epitope on PD1 and displays a markedly slow dissociation rate [78]. Impressive response rates of ~80% and CR rate ranging from 30 to 60% in single-arm phase 2 studies have been reported [78–81]. Both tislelizumab and camrelizumab demonstrated durable responses (median DOR 31.3 and 31.7 months, respectively), but frequent TRAEs and irAEs (45.7% and 98.7%, respectively), the majority of which were grades 1/2 [80–82]. Camrelizumab has a particularly unique toxicity profile with reactive cutaneous capillary endothelial proliferation (RCCEP) seen in 97% of participants in the phase II trial in relapsed Hodgkin lymphoma [82]. This unusual toxicity profile underscores that not all PD1 inhibitors are the same. Whilst the response rates appear impressive, it is worth noting the significant difference in the baseline characteristic of the study population in these trials compared to CheckMate-205 and KEYNOTE-087. Over 80% of these patients had not received a prior ASCT and less than 10% had prior BV. Ethnic difference in response to CPI therapy also cannot be excluded. Head-to-head comparison is ideally needed to see if they are truly more efficacious than CPIs in established use.

## Conclusions

CPIs have greatly expanded the treatment options for patients with R/R cHL, demonstrating efficacy at all points in the disease course. The plethora of recent studies, often small and non-randomised, has raised many questions about how to optimally integrate these drugs into clinical practice. The ability to use CPIs at different stages of the R/R cHL pathway also frequently depends on the licensing status and reimbursement mechanisms within the geographical region of the treating centre. For example, the FDA license for pembrolizumab for adults is for R/R cHL without specifying the number of prior lines of treatment or possible combination partners. In Europe, however, the label specifies monotherapy after 2 more prior lines (or relapse after ASCT). With this in mind and in the absence of a clinical trial, it is the authors’ practise to use pembrolizumab monotherapy or BV after failure of 1st-line relapse chemotherapy, utilising potential chemosensitisation to bridge to ASCT, or nivolumab monotherapy after ASCT (assuming the patient has received prior BV). For those unfit for ASCT, we would use PD1 inhibitors prior to BV generally as it is associated with a longer PFS in a randomised trial. Whilst active when used following an alloSCT, clearly caution needs to be exercised due to the significant incidence of emergent GVHD. Routine use of these agents in this setting would not be recommended. Current data suggest that using CPIs early on, such as at first relapse alone or in combination with chemotherapy or BV, may result in excellent outcomes. Randomised studies, however, are required in order for CPIs to be approved and reimbursed for earlier lines of treatment in multiple countries. Whilst cHL is a relative uncommon disease-making randomised studies challenging, the successful and timely completion of KEYNOTE-204 and AETHERA demonstrate that such studies are feasible with international collaboration.

## Declarations

All reported studies/experiments with human or animal subjects performed by the authors have been previously published and complied with all applicable ethical standards (including the Helsinki declaration and its amendments, institutional/national research committee standards and international/national/institutional guidelines).

## References

[1] Borchmann P, Plütschow A, Kobe C, Greil R, Meissner J, Topp MS (2021). PET-guided omission of radiotherapy in early-stage unfavourable Hodgkin lymphoma (GHSG HD17): a multicentre, open-label, randomised, phase 3 trial. Lancet Oncol.

[2] Kreissl S, Goergen H, Buehnen I, Kobe C, Moccia A, Greil R (2021). PET-guided eBEACOPP treatment of advanced-stage Hodgkin lymphoma (HD18): follow-up analysis of an international, open-label, randomised, phase 3 trial. Lancet Haematol.

[3] André MPE, Girinsky T, Federico M, Reman O, Fortpied C, Gotti M (2017). Early positron emission tomography response-adapted treatment in stage I and II Hodgkin lymphoma: final results of the randomized EORTC/LYSA/FIL H10 trial. J Clin Oncol.

[4] Johnson P, Federico M, Kirkwood A, Fosså A, Berkahn L, Carella A (2016). Adapted treatment guided by interim PET-CT scan in advanced Hodgkin’s lymphoma. N Engl J Med.

[5] Schmitz N, Pfistner B, Sextro M, Sieber M, Carella AM, Haenel M (2002). Aggressive conventional chemotherapy compared with high-dose chemotherapy with autologous haemopoietic stem-cell transplantation for relapsed chemosensitive Hodgkin’s disease: a randomised trial. Lancet.

[6] Linch DC, Winfield D, Goldstone AH, Moir D, Hancock B, McMillan A (1993). Dose intensification with autologous bone-marrow transplantation in relapsed and resistant Hodgkin’s disease: results of a BNLI randomised trial. Lancet.

[7] Bröckelmann PJ, Müller H, Gillessen S, Yang X, Koeppel L, Pilz V, et al. Clinical outcomes of relapsed and refractory Hodgkin lymphoma patients after contemporary first-line treatment: a German Hodgkin Study Group analysis. Leukemia. 2022;36(3):772–80. 10.1038/s41375-021-01442-8. 10.1038/s41375-021-01442-8 PMC8885415 34628472

[8] Moskowitz CH, Matasar MJ, Zelenetz AD, Nimer SD, Gerecitano J, Hamlin P (2012). Normalization of pre-ASCT, FDG-PET imaging with second-line, non-cross-resistant, chemotherapy programs improves event-free survival in patients with Hodgkin lymphoma. Blood..

[9] Moskowitz AJ, Perales M-A, Kewalramani T, Yahalom J, Castro-Malaspina H, Zhang Z (2009). Outcomes for patients who fail high dose chemoradiotherapy and autologous stem cell rescue for relapsed and primary refractory Hodgkin lymphoma. Br J Haematol.

[10] von Tresckow B, Müller H, Eichenauer DA, Glossmann JP, Josting A, Böll B (2014). Outcome and risk factors of patients with Hodgkin Lymphoma who relapse or progress after autologous stem cell transplant. Leuk Lymphoma.

[11] Arai S, Fanale M, DeVos S, Engert A, Illidge T, Borchmann P (2013). Defining a Hodgkin lymphoma population for novel therapeutics after relapse from autologous hematopoietic cell transplant. Leuk Lymphoma..

[12] Martínez C, Canals C, Sarina B, Alessandrino EP, Karakasis D, Pulsoni A (2013). Identification of prognostic factors predicting outcome in Hodgkin’s lymphoma patients relapsing after autologous stem cell transplantation. Ann Oncol.

[13] Younes A, Gopal AK, Smith SE, Ansell SM, Rosenblatt JD, Savage KJ (2012). Results of a pivotal phase II study of brentuximab vedotin for patients with relapsed or refractory Hodgkin’s lymphoma. J Clin Oncol.

[14] Chen R, Gopal AK, Smith SE, Ansell SM, Rosenblatt JD, Savage KJ (2016). Five-year survival and durability results of brentuximab vedotin in patients with relapsed or refractory Hodgkin lymphoma. Blood..

[15] Ansell SM, Lesokhin AM, Borrello I, Halwani A, Scott EC, Gutierrez M (2015). PD-1 blockade with nivolumab in relapsed or refractory Hodgkin’s lymphoma. N Engl J Med.

[16] Armand P, Shipp MA, Ribrag V, Michot J-M, Zinzani PL, Kuruvilla J (2016). Programmed death-1 blockade with pembrolizumab in patients with classical Hodgkin lymphoma after brentuximab vedotin failure. J Clin Oncol.

[17.•] Chen R, Zinzani PL, Fanale MA, Armand P, Johnson NA, Brice P (2017). Phase II study of the efficacy and safety of pembrolizumab for relapsed/refractory classic Hodgkin lymphoma. J Clin Oncol.

[18.•] Younes A, Santoro A, Shipp M, Zinzani PL, Timmerman JM, Ansell S (2016). Nivolumab for classical Hodgkin’s lymphoma after failure of both autologous stem-cell transplantation and brentuximab vedotin: a multicentre, multicohort, single-arm phase 2 trial. Lancet Oncol.

[19] Chen R, Zinzani PL, Lee HJ, Armand P, Johnson NA, Brice P (2019). Pembrolizumab in relapsed or refractory Hodgkin lymphoma: 2-year follow-up of KEYNOTE-087. Blood..

[20] Armand P, Zinzani PLL, Lee HJ, Johnson N, Brice P, Radford J (2021). Five-year follow-up of Keynote-087: pembrolizumab monotherapy in relapsed/refractory classical Hodgkin lymphoma (R/R cHL). Blood..

[21.•] Armand P, Chen Y-B, Redd RA, Joyce RM, Bsat J, Jeter E (2019). PD-1 blockade with pembrolizumab for classical Hodgkin lymphoma after autologous stem cell transplantation. Blood..

[22] Armand P, Engert A, Younes A, Fanale M, Santoro A, Zinzani PL (2018). Nivolumab for relapsed/refractory classic Hodgkin lymphoma after failure of autologous hematopoietic cell transplantation: extended follow-up of the multicohort single-arm phase II CheckMate 205 trial. J Clin Oncol.

[23] Ansell S, Bröckelmann P, von Keudell G, Lee HJ, Santoro A, Zinzani PL (2021). HL-398: five-year overall survival from the CheckMate 205 study of nivolumab for relapsed or refractory (R/R) classical Hodgkin lymphoma (cHL). Clin Lymphoma Myeloma Leuk.

[24] Younes A, Santoro A, Shipp M, Zinzani PL, Timmerman JM, Ansell S (2016). Nivolumab for classical Hodgkin’s lymphoma after failure of both autologous stem-cell transplantation and brentuximab vedotin: a multicentre, multicohort, single-arm phase 2 trial. Lancet Oncol.

[25] Moskowitz CH, Nademanee A, Masszi T, Agura E, Holowiecki J, Abidi MH (2015). Brentuximab vedotin as consolidation therapy after autologous stem-cell transplantation in patients with Hodgkin’s lymphoma at risk of relapse or progression (AETHERA): a randomised, double-blind, placebo-controlled, phase 3 trial. Lancet..

[26] Herrera AF, Chen L, Nieto Y, Holmberg L, Johnston PB, Mei M (2020). Consolidation with nivolumab and brentuximab vedotin after autologous hematopoietic cell transplantation in patients with high-risk Hodgkin lymphoma. Blood..

[27] Zinzani PL, Lee HJ, Armand P, Johnson N, Brice P, Radford J (2019). Three-year follow-up of Keynote-087: pembrolizumab monotherapy in relapsed/refractory classic Hodgkin lymphoma. Blood..

[28.•] Kuruvilla J, Ramchandren R, Santoro A, Paszkiewicz-Kozik E, Gasiorowski R, Johnson NA (2021). Pembrolizumab versus brentuximab vedotin in relapsed or refractory classical Hodgkin lymphoma (KEYNOTE-204): an interim analysis of a multicentre, randomised, open-label, phase 3 study. Lancet Oncol.

[29] Manson Guillaume, Brice Pauline, Herbaux Charles, Bouabdallah Kamal, Antier Chloé, Poizeau Florence (2020). Efficacy of anti-PD1 re-treatment in patients with Hodgkin lymphoma who relapsed after anti-PD1 discontinuation. Haematologica..

[30] Fedorova LV, Lepik KV, Mikhailova NB, Kondakova EV, Zalyalov YR, Baykov VV (2021). Nivolumab discontinuation and retreatment in patients with relapsed or refractory Hodgkin lymphoma. Ann Hematol.

[31] Leger PD, Rothschild S, Castellanos E, Pillai RN, York SJ, Horn L (2017). Response to salvage chemotherapy following exposure to immune checkpoint inhibitors in patients with non-small cell lung cancer. J Clin Oncol.

[32] Park SE, Lee SH, Ahn JS, Ahn M-J, Park K, Sun J-M (2018). Increased response rates to salvage chemotherapy administered after PD-1/PD-L1 inhibitors in patients with non-small cell lung cancer. J Thorac Oncol.

[33] Carreau NA, Armand P, Merryman RW, Advani RH, Spinner MA, Herrera AF (2020). Checkpoint blockade treatment sensitises relapsed/refractory non-Hodgkin lymphoma to subsequent therapy. Br J Haematol.

[34] Carreau NA, Pail O, Armand P, Merryman R, Advani RH, Spinner MA (2020). Checkpoint blockade treatment may sensitize Hodgkin lymphoma to subsequent therapy. Oncologist.

[35] Merryman RW, Redd RA, Nishihori T, Chavez J, Nieto Y, Darrah JM (2021). Autologous stem cell transplantation after anti-PD-1 therapy for multiply relapsed or refractory Hodgkin lymphoma. Blood Adv.

[36] Moskowitz CH, Walewski J, Nademanee A, Masszi T, Agura E, Holowiecki J (2018). Five-year PFS from the AETHERA trial of brentuximab vedotin for Hodgkin lymphoma at high risk of progression or relapse. Blood..

[37] Santoro A, Mazza R, Pulsoni A, Re A, Bonfichi M, Zilioli VR (2016). Bendamustine in combination with gemcitabine and vinorelbine is an effective regimen as induction chemotherapy before autologous stem-cell transplantation for relapsed or refractory Hodgkin lymphoma: final results of a multicenter phase II study. J Clin Oncol.

[38] Bartlett NL, Niedzwiecki D, Johnson JL, Friedberg JW, Johnson KB, van Besien K (2007). Gemcitabine, vinorelbine, and pegylated liposomal doxorubicin (GVD), a salvage regimen in relapsed Hodgkin’s lymphoma: CALGB 59804. Ann Oncol.

[39] Baetz T, Belch A, Couban S, Imrie K, Yau J, Myers R (2003). Gemcitabine, dexamethasone and cisplatin is an active and non-toxic chemotherapy regimen in relapsed or refractory Hodgkin’s disease: a phase II study by the National Cancer Institute of Canada Clinical Trials Group. Ann Oncol.

[40] Santoro A, Magagnoli M, Spina M, Pinotti G, Siracusano L, Michieli M (2007). Ifosfamide, gemcitabine, and vinorelbine: a new induction regimen for refractory and relapsed Hodgkin’s lymphoma. Haematologica..

[41] Josting A, Rudolph C, Reiser M, Mapara M, Sieber M, Kirchner HH (2002). Time-intensified dexamethasone/cisplatin/cytarabine: an effective salvage therapy with low toxicity in patients with relapsed and refractory Hodgkin’s disease. Ann Oncol.

[42] Moskowitz CH, Nimer SD, Zelenetz AD, Trippett T, Hedrick EE, Filippa DA (2001). A 2-step comprehensive high-dose chemoradiotherapy second-line program for relapsed and refractory Hodgkin disease: analysis by intent to treat and development of a prognostic model. Blood..

[43] Devillier R, Coso D, Castagna L, Brenot Rossi I, Anastasia A, Chiti A (2012). Positron emission tomography response at the time of autologous stem cell transplantation predicts outcome of patients with relapsed and/or refractory Hodgkin’s lymphoma responding to prior salvage therapy. Haematologica..

[44] Moskowitz AJ, Yahalom J, Kewalramani T, Maragulia JC, Vanak JM, Zelenetz AD (2010). Pretransplantation functional imaging predicts outcome following autologous stem cell transplantation for relapsed and refractory Hodgkin lymphoma. Blood..

[45] Moskowitz AJ, Shah G, Schöder H, Ganesan N, Drill E, Hancock H (2021). Phase II trial of pembrolizumab plus gemcitabine, vinorelbine, and liposomal doxorubicin as second-line therapy for relapsed or refractory classical Hodgkin lymphoma. J Clin Oncol.

[46] Bryan L, Casulo C, Allen P, Smith S, Savas H, Karmali R, et al. Pembrolizumab (PEM) added to ICE chemotherapy results in high complete metabolic response rates in relapsed/refractory classic Hodgkin lymphoma (cHL): a multi-institutional phase II trial. In: American Society of Hematology Annual Meeting & Exposition (2021). 2021.

[47] Herrera AF, Chen RW, Palmer J, Tsai N-C, Mei M, Popplewell LL (2019). PET-adapted nivolumab or nivolumab plus ICE as first salvage therapy in relapsed or refractory Hodgkin lymphoma. Blood..

[48] Spaas M, Lievens Y. Is the combination of immunotherapy and radiotherapy in non-small cell lung cancer a feasible and effective approach? Front Med. 2019;6:244. 10.3389/fmed.2019.00244. 10.3389/fmed.2019.00244 PMC6853895 31788476

[49] Moskowitz AJ, Schöder H, Yahalom J, McCall SJ, Fox SY, Gerecitano J (2015). PET-adapted sequential salvage therapy with brentuximab vedotin followed by augmented ifosamide, carboplatin, and etoposide for patients with relapsed and refractory Hodgkin’s lymphoma: a non-randomised, open-label, single-centre, phase 2 study. Lancet Oncol.

[50] Herrera AF, Palmer J, Martin P, Armenian S, Tsai N-C, Kennedy N (2018). Autologous stem-cell transplantation after second-line brentuximab vedotin in relapsed or refractory Hodgkin lymphoma. Ann Oncol.

[51] Chen R, Palmer JM, Martin P, Tsai N, Kim Y, Chen BT (2015). Results of a multicenter phase II trial of brentuximab vedotin as second-line therapy before autologous transplantation in relapsed/refractory Hodgkin lymphoma. Biol Blood Marrow Transplant.

[52] Advani RH, Moskowitz AJ, Bartlett NL, Vose JM, Ramchandren R, Feldman TA (2021). Brentuximab vedotin in combination with nivolumab in relapsed or refractory Hodgkin lymphoma: 3-year study results. Blood..

[53] Cole P, Mauz-Korholz C, Mascarin M, Michel G, Cooper S, Beishuizen A, et al. Nivolumab and brentuximab vedotin (BV)-based, response‐adapted treatment in children, adolescents, and young adults (CAYA) with standard-risk relapsed/refractory classical Hodgkin lymphoma (R/R cHL): primary analysis. In: ASCO Annual Meeting. 2020.

[54] Armand P, Kuruvilla J, Michot J-M, Ribrag V, Zinzani PL, Zhu Y (2020). KEYNOTE-013 4-year follow-up of pembrolizumab in classical Hodgkin lymphoma after brentuximab vedotin failure. Blood Adv.

[55] Rashidi A, Ebadi M, Cashen AF (2016). Allogeneic hematopoietic stem cell transplantation in Hodgkin lymphoma: a systematic review and meta-analysis. Bone Marrow Transplant.

[56] el Cheikh J, Massoud R, Abudalle I, Haffar B, Mahfouz R, Kharfan-Dabaja MA (2017). Nivolumab salvage therapy before or after allogeneic stem cell transplantation in Hodgkin lymphoma. Bone Marrow Transplant.

[57] Armand P, Zinzani PL, Collins GP, Cohen JB, Halwani AS, Carlo-Stella C (2016). Outcomes of allogeneic hematopoietic stem cell transplantation (HSCT) after treatment with nivolumab for relapsed/refractory Hodgkin lymphoma. Blood..

[58] Dada R, Usman B (2019). Allogeneic hematopoietic stem cell transplantation in r/r Hodgkin lymphoma after treatment with checkpoint inhibitors: feasibility and safety. Eur J Haematol..

[59] Merryman RW, Kim HT, Zinzani PL, Carlo-Stella C, Ansell SM, Perales M-A (2017). Safety and efficacy of allogeneic hematopoietic stem cell transplant after PD-1 blockade in relapsed/refractory lymphoma. Blood..

[60] Beköz H, Karadurmuş N, Paydaş S, Türker A, Toptaş T, Fıratlı Tuğlular T (2017). Nivolumab for relapsed or refractory Hodgkin lymphoma: real-life experience. Ann Oncol.

[61] Herbaux C, Gauthier J, Brice P, Drumez E, Ysebaert L, Doyen H (2017). Efficacy and tolerability of nivolumab after allogeneic transplantation for relapsed Hodgkin lymphoma. Blood..

[62] Paul S, Zahurak M, Luznik L, Ambinder RF, Fuchs EJ, Bolaños-Meade J (2020). Non-myeloablative allogeneic transplantation with post-transplant cyclophosphamide after immune checkpoint inhibition for classic Hodgkin lymphoma: a retrospective cohort study. Biol Blood Marrow Transplant.

[63] Ito A, Kim S-W, Matsuoka K-I, Kawakita T, Tanaka T, Inamoto Y (2020). Safety and efficacy of anti-programmed cell death-1 monoclonal antibodies before and after allogeneic hematopoietic cell transplantation for relapsed or refractory Hodgkin lymphoma: a multicenter retrospective study. Int J Hematol.

[64] Haverkos BM, Abbott D, Hamadani M, Armand P, Flowers ME, Merryman R (2017). PD-1 blockade for relapsed lymphoma post-allogeneic hematopoietic cell transplant: high response rate but frequent GVHD. Blood..

[65] Martínez C, Gayoso J, Canals C, Finel H, Peggs K, Dominietto A (2017). Post-transplantation cyclophosphamide-based haploidentical transplantation as alternative to matched sibling or unrelated donor transplantation for Hodgkin lymphoma: a registry study of the Lymphoma Working Party of the European Society for Blood and Marrow Transplantation. J Clin Oncol.

[66] Schoch LK, Cooke KR, Wagner-Johnston ND, Gojo I, Swinnen LJ, Imus P (2018). Immune checkpoint inhibitors as a bridge to allogeneic transplantation with posttransplant cyclophosphamide. Blood Adv.

[67] Nieto JC, Roldán E, Jiménez I, Fox L, Carabia J, Ortí G (2020). Posttransplant cyclophosphamide after allogeneic hematopoietic cell transplantation mitigates the immune activation induced by previous nivolumab therapy. Leukemia..

[68] Sureda A, Robinson S, Canals C, Carella AM, Boogaerts MA, Caballero D (2008). Reduced-intensity conditioning compared with conventional allogeneic stem-cell transplantation in relapsed or refractory Hodgkin’s lymphoma: an analysis from the Lymphoma Working Party of the European Group for Blood and Marrow Transplantation. J Clin Oncol.

[69] Sureda A, Canals C, Arranz R, Caballero D, Ribera JM, Brune M (2012). Allogeneic stem cell transplantation after reduced intensity conditioning in patients with relapsed or refractory Hodgkin’s lymphoma. Results of the HDR-ALLO study - a prospective clinical trial by the Grupo Español de Linfomas/Trasplante de Médula Osea (GEL/TAMO) and the Lymphoma Working Party of the European Group for Blood and Marrow Transplantation. Haematologica..

[70] Robinson SP, Sureda A, Canals C, Russell N, Caballero D, Bacigalupo A (2009). Reduced intensity conditioning allogeneic stem cell transplantation for Hodgkin’s lymphoma: identification of prognostic factors predicting outcome. Haematologica..

[71] Armand P, Kim HT, Sainvil M-M, Lange PB, Giardino AA, Bachanova V (2016). The addition of sirolimus to the graft-versus-host disease prophylaxis regimen in reduced intensity allogeneic stem cell transplantation for lymphoma: a multicentre randomized trial. Br J Haematol.

[72] Armand P, Kim HT, Ho VT, Cutler CS, Koreth J, Antin JH (2008). Allogeneic transplantation with reduced-intensity conditioning for Hodgkin and non-Hodgkin lymphoma: importance of histology for outcome. Biol Blood Marrow Transplant.

[73] Herbaux C, Merryman R, Devine S, Armand P, Houot R, Morschhauser F (2018). Recommendations for managing PD-1 blockade in the context of allogeneic HCT in Hodgkin lymphoma: taming a necessary evil. Blood..

[74] Manson G, Herbaux C, Brice P, Bouabdallah K, Stamatoullas A, Schiano J-M (2018). Prolonged remissions after anti-PD-1 discontinuation in patients with Hodgkin lymphoma. Blood..

[75] Nie J, Wang C, Liu Y, Yang Q, Mei Q, Dong L (2019). Addition of low-dose decitabine to anti-PD-1 antibody camrelizumab in relapsed/refractory classical Hodgkin lymphoma. J Clin Oncol.

[76] Falchi L, Sawas A, Deng C, Amengual JE, Colbourn DS, Lichtenstein EA (2016). High rate of complete responses to immune checkpoint inhibitors in patients with relapsed or refractory Hodgkin lymphoma previously exposed to epigenetic therapy. J Hematol Oncol.

[77] Herrera AF, Chen L, Popplewell LL, Budde LE, Mei M, Armenian SH (2019). Preliminary results from a phase I trial of pembrolizumab plus vorinostat in patients with relapsed or refractory diffuse large B-cell lymphoma, follicular lymphoma, and Hodgkin lymphoma. Blood..

[78] Hong Y, Feng Y, Sun H, Zhang B, Wu H, Zhu Q (2021). Tislelizumab uniquely binds to the CC’ loop of PD-1 with slow-dissociated rate and complete PD-L1 blockage. FEBS Open Bio..

[79] Shi Y, Su H, Song Y, Jiang W, Sun X, Qian W (2019). Safety and activity of sintilimab in patients with relapsed or refractory classical Hodgkin lymphoma (ORIENT-1): a multicentre, single-arm, phase 2 trial. Lancet Haematol..

[80] Song Y, Gao Q, Zhang H, Fan L, Zhou J, Zou D, et al. Tislelizumab for relapsed/refractory classical Hodgkin lymphoma: 3-year follow-up and correlative biomarker analysis. Clin Cancer Res. 2022;28(6):1147–56. 10.1158/1078-0432.CCR-21-2023. 10.1158/1078-0432.CCR-21-2023 PMC9365351 34716199

[81] Song Y, Gao Q, Zhang H, Fan L, Zhou J, Zou D (2020). Treatment of relapsed or refractory classical Hodgkin lymphoma with the anti-PD-1, tislelizumab: results of a phase 2, single-arm, multicenter study. Leukemia..

[82] Wu J, Song Y, Chen X, Lin T, Cao J, Liu Y, et al. Camrelizumab for relapsed or refractory classical Hodgkin lymphoma: extended follow-up of the multicenter, single-arm, Phase 2 study. Int J Cancer. 2022;150(6):984–92. 10.1002/ijc.33852. 10.1002/ijc.33852 34674396

